# Open Science Within Pediatric Medical and Mental Health Systems: Practical Considerations for Behavioral Health Researchers

**DOI:** 10.3389/fpsyg.2022.856949

**Published:** 2022-03-03

**Authors:** Robyn E. Metcalfe

**Affiliations:** Department of Counseling, Family and Human Services, University of Oregon, Eugene, OR, United States

**Keywords:** research methods, open science, reproducibility, preregistration, pediatric psychology

## Introduction

The open science movement is an epistemological movement toward increasing accessibility of data and research processes in order to improve the quality and reproducibility of science (Hesse, 2018). A number of practices have been proposed in association with open science, including journal clubs, systems for project workflow, sharing code, sharing data, use of preprints, pre-registering studies, open-access publishing, an increased focus on statistical power, and greater transparency in data analysis documentation (Allen and Mehler, 2019; Kathawalla et al., 2021). In particular, researchers promoting open science practices focus on the benefits to scientific inquiry by improving reproducibility, improving public access to scientific findings, and allowing for more active collaboration and building on previous work, among other benefits (Hesse, 2018).

As technology increasingly improves connection between researchers, the pressure to engage in specific open-science practices, such as sharing raw data with other researchers, has increased. Still, data sharing in psychological research remains relatively rare due to a range of potential barriers (Houtkoop et al., 2018). Researchers identify practical concerns about the process of data-sharing, concerns about anonymity of participants, and concerns about being credited on subsequent research projects as specific concerns related to data-sharing (Cheah et al., 2015).

Further, implementation of open science practices has been uneven across settings, with pediatric medical and mental health system-based research lagging behind other psychology research contexts. Fewer than 10% of studies from these hospital-based settings engage in even low-stakes open science practices such as publishing supplemental code or promptly publishing results in clinical trials registries (Sixto-Costoya et al., 2020; Kadakia et al., 2021), let alone high-stakes or potentially difficult-to-implement practices. This problem is compounded by low standards for adherence to best practices by journals in these fields (Gardener et al., 2022). Despite clear potential for clinical benefit to pediatric populations when open science practices are utilized (Rubinstein et al., 2020), there has been little exploration or published discussion about how barriers are perceived by researchers in these systems or discussion about how previously established solutions to barriers might apply to these researchers.

Children and families within systemic pediatric medical or mental health systems are considered sensitive populations, deserving of particular ethical consideration in research and clinical processes (American Psychological Association, 2017), and their behavioral health data is often sensitive and/or identifiable. Goodie et al. (2013) advise that, for sensitive populations, researchers and other stakeholders should consider the balance between providing high quality, patient-centered services while collecting sound scientific data and decreasing the likelihood of adverse outcomes. Further, grant-funded research in pediatric medical and mental health systems is often costly and high-stakes, leading to particularly robust discussion of risk-management for participants in these settings (Wendler, 2006). These concerns about open science practices by behavioral health researchers are legitimate and worthy of serious consideration. Considerations for engaging in open science practices in these settings may be different than in other psychological research. Due to the potential need for additional protections, consideration of open science methods in the context of these populations is warranted.

## Factors

### Consideration: Protection of Participant Privacy

Concerns about participant welfare rightfully concern behavioral health researchers in pediatric settings who are considering open science practices. Sharing research data may increase the risk of compromising confidential information about participants. While many of these risks are preventable, adequate protection requires both preventative planning and clear informed consent by participants (Alter and Gonzalez, 2018). Some research, such as longitudinal studies or research that includes geographic tracking, may be particularly difficult to de-identify without compromising the research value of the dataset. Medical and mental health data has long been known to be at particular risk of re-identifying participants when combined with basic demographic data (Sweeney, 2000). In pediatric settings, where demographic information may include both children and their parents or guardians, the potential to re-identify participants is additionally heightened.

Data collected in medical or mental health settings also likely falls under other legislation, such as the Health Insurance Portability and Accountability Act (HIPAA) in the United States, the General Data Protection Regulation (GDPR) in the European Union, or the Personal Information Protection and Electronic Documents Act (PIPEDA) in Canada, and any data-sharing plan must consider this explicitly.

#### Actions for Researchers

Of course, researchers should ensure that sufficient processes are in place to ensure participant safety and appropriately document this with their Institutional Review Board. Researchers should engage in robust risk-management strategies regardless of whether or not they intend to share their data (e.g., data encryption for personal health data, splitting datasets to separate identifiable information from ID variables, etc.). Depending on the types of information gathered by researchers, partial datasets may be realistic for some projects, with only non-sensitive data available for data sharing. Example repositories include Open Science Framework (OSF), Mendeley Data, Figshare, and Zenodo.

However, in the context of sensitive personal health data that makes data sharing infeasible, researchers may also benefit from a selective use of open science practices. For example, in situations where data sharing is not feasible (e.g., due to patient confidentiality concerns), researchers may still choose to engage with other open science practices such as preregistration, reproducible code, and preprints. Additionally, publishing adequate summary statistics provides readers greater opportunity to evaluate a research paper, particularly in contexts where original datasets have not be shared. For example, in addition to means and standard deviations, a researcher might choose to publish variance-covariance matrices of all variables in their work. Researchers may also choose to publish their statistical script alongside research papers to improve reader confidence in their results and facilitate researcher learning (Mair, 2016).

Kathawalla et al. (2021) identify the least difficult to most difficult open science practices as: (1) Starting an open science journal club, (2) Developing an open science-friendly project workflow, (3) Posting pre-prints, (4) Using reproducible code, (5) Sharing data, (6) Transparent manuscript writing, (7) Preregistering research, and (8) Publishing registered reports. Given that this list was not designed with protected health data in mind, research in pediatric medical and mental health systems, this difficulty order may be somewhat different, with data sharing likely causing the most significant concern, due to the potentially sensitive nature of these datasets. Even in cases where no data sharing is feasible, researchers can likely engage in some of the other open science practices endorsed by Kathawalla et al. (2021).

### Consideration: Propriety of Information

Data collected in pediatric medical and mental health settings are often collected at great monetary and temporal expense. Researchers in these settings may intend to produce a large number of publications with a single dataset and worry that procedures such as data sharing may result in reduced ability to maximize their collected materials for their own research output. Concerns about the ability to adequately utilize one's own dataset, procedure, or planned analyses before opening it up to other researchers are valid.

#### Actions for Researchers

In addition to choosing some of the other open science practices discussed above, researchers may consider using project management tools that enable timelocks (e.g., preregistering a hypothesis and not publicly releasing it until there has been sufficient time to complete all relevant work) to avoid the possibility of getting “scooped,” a colloquial term referring to instances where one researcher publishes a novel project in advance of another researcher, who was already working on this idea. If data sharing is possible, it may be reasonable to require that researchers request access in order to access the full dataset. In these cases, sharing variables or codebook material may be more feasible. Notably, there are likely advantages of data sharing, when feasible to do so, for overall research impact. For example, linking data in a repository increases researchers' citation count by 25% (Colavizza et al., 2020). Researchers may also wish to license their data, code, and materials to ensure that it is used appropriately (e.g., a Creative Commons license).

### Consideration: Data Ownership and Permissions

Ownership and legal issues may disrupt the possibility of data sharing even among willing participants. In many cases, the grantee for ownership of research data is the university or research hospital, not the individual researcher (Alter and Gonzalez, 2018). Other times, research may be published that is clearly not within the purview of the project leader. For example, a secondary data analysis by a graduate student, completed with permission from the data steward, is almost certain to be derived from a dataset that is not the intellectual property of the student. In these cases, a researcher may be unsure of their ability to share their data, or simply unable to do so due to restrictions imposed by the data owner.

Previously-collected data in pediatric settings may also lack adequate informed consent procedures to facilitate data-sharing, or complications may arise in creating pathways for sharing sensitive or protected information in these settings. These concerns may be more likely to come up for large scale studies operating out of medical centers within major pediatric health systems.

#### Actions for Researchers

Researchers in leadership roles may find value in drafting guidelines for open science practices within their organization in collaboration with their research ethics team to improve access to data sharing options. For example, if researchers have historically used consents that prohibit data sharing, a relatively common practice in large healthcare facilities, choosing a more flexible option with informed consent of participants may allow for more open science-related workability in future projects.

Importantly, Campbell et al. (2019) highlight the complications of setting up adequate informed consent procedures for highly sensitive information or for populations that have historically been wronged by psychological or medical researchers. They recommend a tiered consent approach to allow parents or guardians to choose the level of data sharing that feel comfortable with. Notably, children in pediatric settings are not able to offer informed consent to participation in research. Based on developmental stage, children may be able to provide assent in conjunction with parental consent. Thus, child health and mental health data deserves additional considerations related to consent when children reach the age of majority, such as policies around re-contacting to establish consent for data-sharing when children become adults (Brothers et al., 2014). Despite these potential complications, setting up standard procedures to facilitate the conversations around open science practices can improve the reproducibility of future research.

## Discussion

Generally, the benefits of open science practices have the potential to be robust. However, legitimate concerns may limit the ability of some researchers operating within healthcare settings to engage in certain practices such as data sharing. Although steps can be taken to encourage data sharing when feasible, importantly, there are a range of potential actions in addition to data-sharing that researchers can take to improve both the accessibility and reproducibility of science. This article aims to encourage interested but hesitant researchers in pediatric medical and mental health systems to consider manageable steps in the direction of open science.

In particular, researchers are encouraged to:

1. Explicitly consider which open science practices are feasible for their project and specific datasets with large health and mental health systems.
2. Proactively plan for large scale research trials to ensure rigorous data reporting standards while balancing the rights of children and families who participate in research.
3. Connect with peers to create a culture that encourages open science within pediatric-focused institutions including active supports for researchers.

Future research should examine setting-specific barriers or hesitations to engaging with open science practices as well as facilitators and the impacts of potential interventions (e.g., creating an open science journal club) on the reproducibility of research within institutions.

## Author Contributions

The author confirms being the sole contributor of this work and has approved it for publication.

## Conflict of Interest

The author declares that the research was conducted in the absence of any commercial or financial relationships that could be construed as a potential conflict of interest.

## Publisher's Note

All claims expressed in this article are solely those of the authors and do not necessarily represent those of their affiliated organizations, or those of the publisher, the editors and the reviewers. Any product that may be evaluated in this article, or claim that may be made by its manufacturer, is not guaranteed or endorsed by the publisher.
